# Eating more sardines instead of fish oil supplementation: Beyond omega-3 polyunsaturated fatty acids, a matrix of nutrients with cardiovascular benefits

**DOI:** 10.3389/fnut.2023.1107475

**Published:** 2023-04-14

**Authors:** Heitor O. Santos, Theresa L. May, Allain A. Bueno

**Affiliations:** ^1^School of Medicine, Federal University of Uberlandia (UFU), Uberlandia, Minas Gerais, Brazil; ^2^School of Science and the Environment, University of Worcester, Worcester, United Kingdom

**Keywords:** sardines, fish oil, omega-3, lipids, cardiovascular disease, calcium

## Abstract

Omega-3 polyunsaturated fatty acids (n-3 PUFA) play a significant role in the prevention and management of cardiometabolic diseases associated with a mild chronic pro-inflammatory background, including type 2 diabetes, hypertension, hypertriglyceridaemia, and fatty liver disease. The effects of n-3 PUFA supplements specifically, remain controversial regarding reducing risks of cardiovascular events. n-3 PUFA supplements come at a cost for the consumer and can result in polypharmacy for patients on pharmacotherapy. Sardines are a well-known, inexpensive source of n-3 PUFA and their consumption could reduce the need for n-3 PUFA supplementation. Moreover, sardines contain other cardioprotective nutrients, although further insights are crucial to translate a recommendation for sardine consumption into clinical practice. The present review discusses the matrix of nutrients contained in sardines which confer health benefits for cardiometabolism, beyond n-3 PUFA. Sardines contain calcium, potassium, magnesium, zinc, iron, taurine, arginine and other nutrients which together modulate mild inflammation and exacerbated oxidative stress observed in cardiovascular disease and in haemodynamic dysfunction. In a common serving of sardines, calcium, potassium, and magnesium are the minerals at higher amounts to elicit clinical benefits, whilst other nutrients are present in lower but valuable amounts. A pragmatic approach towards the consumption of such nutrients in the clinical scenario should be adopted to consider the dose–response relationship effects on physiological interactions. As most recommendations currently available are based on an indirect rationale of the physiological actions of the nutrients found in sardines, randomised clinical trials are warranted to expand the evidence on the benefits of sardine consumption.

**Graphical Abstract:**
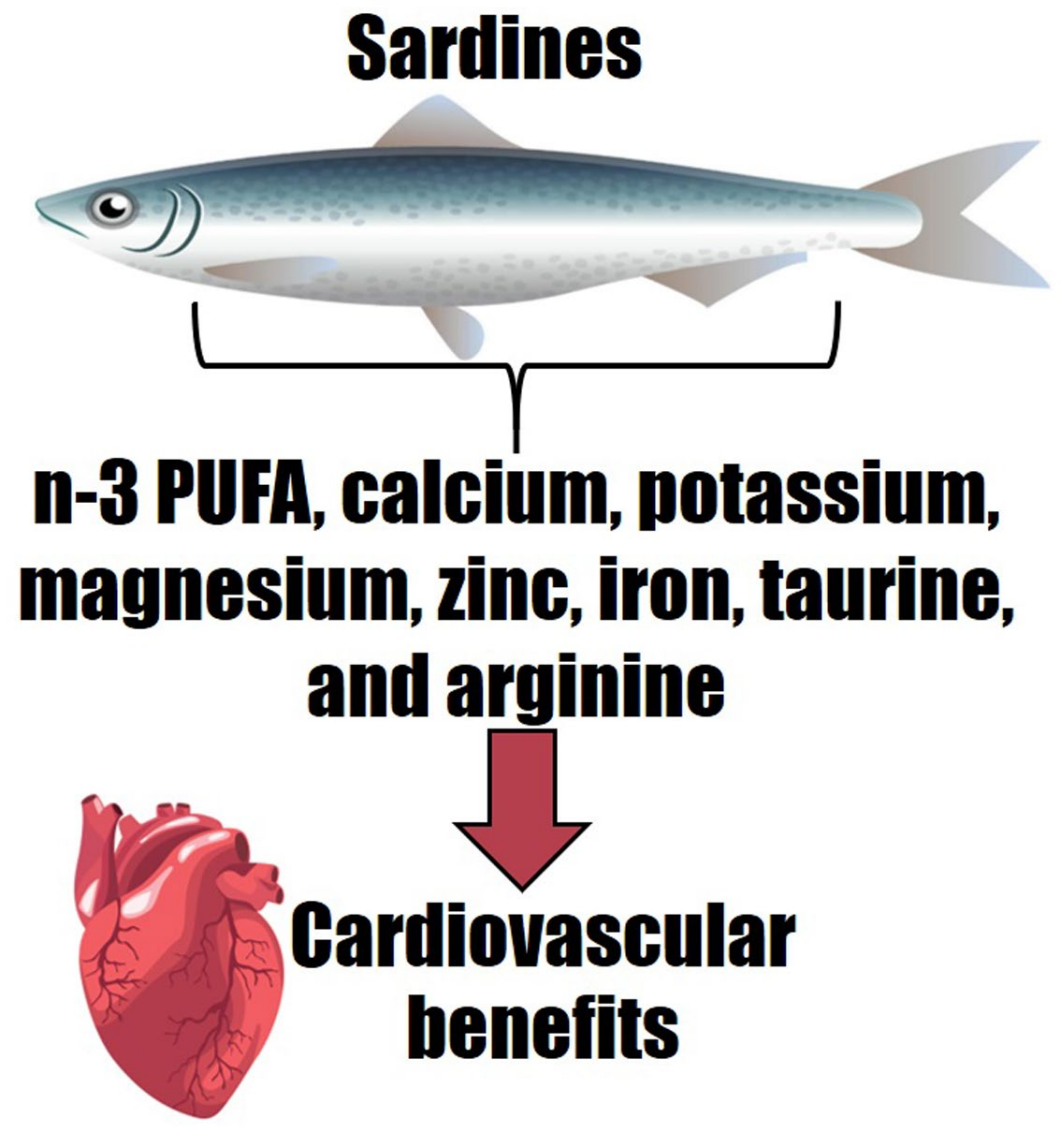


## 1. Introduction

Omega-3 polyunsaturated fatty acids (n-3 PUFA) are recognised as functional nutrients with multifaceted effects in preventing and managing cardiometabolic diseases with pro-inflammatory backgrounds, such as type 2 diabetes (T2D), hypertension, hypertriglyceridaemia, and fatty liver disease (1–5). Extensive research conducted in the past decades has confirmed the beneficial effects of fish oil supplementation as a source of n-3 PUFA, mainly eicosapentaenoic acid (EPA) and docosahexaenoic acid (DHA) (6–9).

Long-term adherence to fish oil supplementation however is currently a matter for debate. With large segments of the general population, particularly older age groups, relying on prescribed medication for a range of health conditions, such a valuable nutritional approach when mismanaged can result in polypharmacy (10–13).

The proposed therapeutic dosage for fish oil supplementation (EPA + DHA ≥ 3 g/d) can lead to a higher intake of capsules, posing a challenge in a chronic treatment plan. Moreover, side-effects including eructation related to fishy taste and nausea are common (14). The gastric capacity of the individual cannot be neglected; as a sufficient volume of water must be ingested with the capsules to allow efficient gastric mixture, aged patients and those suffering from gastroesophageal reflux disease may experience difficulty in complying with the ideal dosage both on fasting or postprandially.

A range of foodstuffs are important sources of n-3 PUFA, but unlike fish oil supplementation, an impasse remains when translating therapeutic n-3 PUFA dosage to actual food intake due to the broad range and varied concentration across dietary sources of n-3 PUFA (15).

Oily fish is the most relevant dietary source of n-3 PUFA (16, 17); however, the n-3 PUFA content substantially varies depending on the fish (18). Sardines are an inexpensive type of fish with high n-3 PUFA content (19, 20). Moreover, sardines are a source of many essential nutrients, including minerals, vitamins and amino acids, whose considerable amounts may improve clinical outcomes in patients affected by cardiometabolic diseases (21, 22).

Guidelines suggest sardine consumption as a source of dietary n-3 PUFA; nevertheless, most evidence available is based on observational studies (23, 24). Despite a lack of scientific robustness, a few randomised clinical trials (RCTs) investigating the effects of sardine consumption have been published recently (25, 26), but the need to infer the current clinical applicability for practitioners remains. The aims of the present study were (1) to appraise existing RCTs that have investigated sardine consumption, providing an indirect comparison with other dietary sources of n-3 PUFA, in order to draw pragmatic conclusions, and (2) to expand the clinical rationale related to functional nutrients contained in sardines that can elicit cardiovascular benefits.

## 2. Micronutrients from sardines in cardiovascular disease

Sardines are not only a relevant source of n-3 PUFA, but also a source of calcium, presenting a higher amount of both nutrients compared to other fish (Table 1). The calcium content in 100 g of sardines is equivalent to the amount found in ~ 400 ml of milk (27), thus being an alternative for individuals who cannot tolerate dairy products or milk at that volume, and also an option for those in need of complementing their total calcium intake in general. More specifically, 100 g sardines offer 382 mg/d of calcium, which accounts for 38% of the recommended dietary allowance (RDA) (1,000 mg/d in general) (28).

**Table 1:** Nutrition facts of sardines compared with other popular fish (values per 100 g).

Nutrition Facts	Sardines, cooked (FDC ID: 1098981)	Sardines with tomato-based sauce (FDC ID: 1099277)	Tuna, canned, oil pack (FDC ID: 1099041)	Salmon, baked or broiled (FDC ID: 1098966)	Tilapia, cooked (FDC ID: 175177)	Cod, smoked (FDC ID: 1098818)
Energy, kcal	208	185	198	164	128	108
Protein, g	24.6	20.9	29.1	25.7	26.2	23.9
Total lipid, g	11.5	10.5	8.2	5.9	2.7	0.6
Calcium, mg	382	240	13	9	14	13
Iron, mg	2.9	2.3	1.4	0.5	0.7	0.3
Magnesium, mg	39	34	31	34	34	31
Phosphorus, mg	490	366	311	328	204	440
Potassium, mg	397	341	207	461	380	368
Sodium, mg	307	414	416	415	56	625
Zinc, mg	1.3	1.4	0.9	0.5	0.41	0.48
Copper, mg	0.186	0.272	0.071	0.079	0.075	0.03
Selenium, μg	52.7	40.6	76.0	39.4	54.4	35.9
Niacin, mg	5.3	4.2	12.4	9.5	4.7	1.6
Vitamin B-12, μg	8.9	9	2.2	4.7	1.9	2.8
Vitamin A, RAE, μg	32	34	23	40	0	3
Lycopene, μg	0	1,398	0	0	0	0
Vitamin E, mg	0	1.38	0.87	0.50	0.79	0.84
Vitamin D (D2 + D3), μg	4.8	4.8	6.7	13.7	3.7	0.8
Saturated fat, g	1.5	2.7	1.5	1.0	0.9	0.1
PUFA 2:5 *n*–3 (EPA), g	0.473	0.532	0.027	0.228	0.005	0.053
PUFA 22:5 *n*–3 (DPA), g	0	0.061	0	0.059	0.06	0.006
PUFA 22:6 *n*–3 (DHA), g	0.509	0.864	0.101	0.418	0.13	0.15
Cholesterol, mg	142	61	18	58	57	74

Adapted from the United States Department of Agriculture (USDA) database (29).

Calcium is a fundamental nutrient in cardiovascular biochemical pathways (30), as evidenced by its major role as signalling ion and paramount for myocardial contractility (31). Calcium is indispensable for bone health (32), and approximately 99% of the calcium bodily content is contained in bone tissue, with the remaining 1% stored in other tissues and with varied but similarly important biochemical roles. Aside from calcium, sardines are an important source of magnesium, phosphorus, and vitamin D, which collectively contribute to bone metabolism (33, 34) and are similarly important for cardiovascular health (35–37).

Sardines, as a dietary source of n-3 PUFA, have shown benefits for blood pressure (BP) and lipid profile normalisation (38, 39). Moreover, sardines are a source of potassium, magnesium, and zinc, which are nutrients with observed BP lowering properties, as well as niacin and zinc, which in turn are candidates for improving circulating levels of lipids and lipoproteins (40–44).

Given that the overall dietary potassium intake corresponds to 1.6 to 5.9 g/d (45), sardines can be considered a relevant source of potassium, at 490 mg in a 100 g serving (Table 1). Adequate potassium intake can not only normalise elevated BP by increasing natriuresis when the cause of hypertension is high sodium levels, it also favours optimal endothelial function and nitric oxide release (46). Furthermore, endothelial hyperpolarization induced by the cell membrane potassium channels decreases cytosolic smooth muscle cell calcium, with subsequent vasodilatation induction and ultimately decreasing BP (46). Through similar biochemical actions, potassium can attenuate sympathetic activity, triggering vascular muscle relaxation and hence lowering elevated BP (46). Considering such relevant actions, current guidelines consider the increase in dietary potassium as a Grade A recommendation to reduce BP (47). A dose–response meta-analysis found an inverse association between potassium intake and stroke risk at a dose of ~ 3,500 mg/d, which may be reached by a balanced diet (48). Overall, sardines can be deemed as a food candidate to contribute to optimal dietary potassium intake (49).

Sardines contain a considerable amount of iron (2.9 mg/100 g), higher than other commonly consumed types of fish (Table 1). Sardine iron content is comparable to that of meat, the most popular source of iron worldwide (50). Thus, sardine consumption is an option to reach the RDA for iron, 8 mg/d for all age groups (51), and particularly helpful for those who do not eat meat. Given the well-established association between iron-deficiency anaemia and cardiovascular diseases, iron intake should not be neglected when managing cardiovascular patients. Iron deficiency is a contributing factor for negative outcomes in patients with coronary artery disease, heart failure and pulmonary hypertension, with the elderly and those with coexisting chronic diseases being more vulnerable (52).

Iron supplementation may be necessary in specific cases, but at the same time sardine consumption could be an ideal option to be included in the diet plan. On the other hand, excessive iron intake must be avoided as excess iron is detrimental to cardiovascular health due to its very high reactivity and increased iron-catalysed free radical-mediated oxidative stress. Iron intake derived from sardine consumption, as well as from other iron-containing foods, do not cause such undesired effects, unlike iron oral supplementation which can more easily lead to iron overload and exacerbate haemochromatosis when mismanaged (53, 54).

## 3. Functional amino acids across sardines vs. supplemental therapies

Sardines are an important source of amino acids, including arginine, taurine and others, which play fundamental roles in cardiometabolism, not only as structural biomolecules but also as modulators of antioxidant systems and vascular function (55–57). Regarding arginine, although it is a conditionally essential amino acid, it is vital for BP stability and general vascular health, serving as a substrate in the endothelium-derived nitric oxide synthetic pathway, thereby reducing systemic BP (58, 59). Moreover, arginine can improve renovascular hypertension *via* the renin-angiotensin system by increasing nitric oxide synthesis and reducing angiotensin II (60).

In general, the average intake of arginine at populational level is close to 4 g daily. For example, the mean arginine intake for the US adult population is approximately 4.4 g/d, with 25% of people consuming less than 2.6 g/d (61). In Finnish men aged 42–60 years, an average arginine intake of 3.8 mg/d was reported (62), while preliminary reporting from the Zutphen Elderly Study, a branch of the Seven Countries Study, found in Dutch men aged 64–84 years a mean arginine intake of 4.4 g/d (63). However, due to the observational nature of their design, those studies do not support the hypothesis that higher arginine intake lowers BP or coronary heart disease mortality (62, 63). Nonetheless, clinical findings suggest that arginine supplementation can reduce BP, but at an oral dosage of 4 to 24 g/d for 2 to 24 weeks, as reported in a meta-analysis of RCT describing reductions of 5.39 mmHg (95% CI −8.54 to −2.25) and 2.66 mmHg (95% CI −3.77 to −1.54) in systolic and diastolic BP, respectively (64).

Despite the lack of robust clinical findings on the relationship between arginine intake and cardiovascular outcomes, habitual sardine consumption can contribute to overall arginine intake. According to the United States Department of Agriculture (USDA) database (29), the arginine content in cooked sardines is 1.47 g/100 g (FDC ID: 175139). However, it must be noted that sardine arginine content is not higher than that of tuna (1.79 g/100 g; FDC ID: 173707), salmon (1.72 g/100 g; FDC ID: 173692) or beef (1.71 g/100 g; FDC ID: 168720) (29).

Taurine is an amino sulfonic acid with a range of biochemical roles, and it has been observed that its antioxidant activity modulates the cardiovascular system, leading to clinical benefits including BP normalisation and amelioration in lipid and glycaemic indices (65–67). Taurine itself does not scavenge reactive oxygen species such as superoxide anion, hydroxyl radical, and hydrogen peroxide (68), but 5-taurinomethyluridine, a derivative of taurine conjugation with uridine on mitochondrial tRNA, modulates mitochondrial protein synthesis and enhances electron transport chain activity (69), protecting the mitochondrion against excessive reactive oxygen and nitrogen species (70, 71). Redox modulation is favourable to blood vessels, but taurine may also lower BP and improve vascular function through additional pathways, for example suppressing renin–angiotensin–aldosterone system activity and augmenting kallikrein activity in blood and peripheral tissues (72).

Cholesterol-lowering properties have been attributed to taurine, including upregulation of the hepatic low-density lipoprotein receptor (LDLR) and facilitation of the binding of LDL particles to LDLR, as well as reduction of hepatic activity of acyl-CoA:cholesterol acyltransferase (ACAT) and enhancement of cholesterol 7alpha-hydroxylase activity (73). ACAT is a membrane-bound enzyme that utilises long-chain fatty acyl-CoA and cholesterol as substrates to synthetise cholesteryl ester, whilst cholesterol 7alpha-hydroxylase catalyses the initial step in cholesterol catabolism and bile acid synthesis (74, 75). Moreover, taurine contributes to glucose homeostasis by modulating gene expression that is essential for glucose-stimulated insulin secretion (i.e., sulfonylurea receptor-1, glucokinase, Glut-2, proconvertase, and pancreas duodenum homeobox-1) and increasing insulin-stimulated tyrosine phosphorylation of the insulin receptor in skeletal muscle and liver, consequently ameliorating peripheral insulin sensitivity and improving overall glycaemic profile (76).

Sardines contain 147 mg taurine/100 g (25), which is not higher than taurine content in tuna (332 mg), beef (344 mg), pork (489 mg), and dark meat chicken (1,355 mg)/100 g (77, 78). As expected, interventions based on a sardine-rich diet increase taurine intake when compared to control groups. For instance, adding 100 g of sardines 5 days a week to a diet plan resulted in a significant increase in taurine intake by 118.5 mg/d, whilst adding 200 g of sardine weekly increased taurine intake by 43.2 mg/d (25, 26). Although sardines are a relevant source of taurine, its content is not compatible with the supplementation dosage that is necessary to elicit clinical benefits; therefore, caution should be exercised not to extrapolate the aforementioned findings. A meta-analysis of RCTs found that the taurine dosage that triggered a mean decrease of ~3 mmHg in both SBP and DBP was 1–6 g/d for 1 day to 12 weeks (79).

## 4. Mercury and selenium: The ‘yin and yang’

Fish consumption is recommended as part of a healthy diet, but inherent concerns remain regarding mercury-contaminated fish and the toxicological roles of mercury in cardiovascular and neural diseases (80, 81). As a potent and seemingly irreversible inhibitor of the key antioxidant selenoenzymes, mercury hinders the actions of glutathione peroxidases and thioredoxin reductases by binding to selenocysteine at the active site of these enzymes, resulting in impaired antioxidant protection (81). The well-documented toxicological effects of mercury can be partially mitigated by selenium intake, which is incorporated to synthesize new selenoenzymes (81). If this proposed rationale is correct, it is worth noting that sardines contain considerable selenium content and, unlike tuna, are placed at a mid-trophic level, near the base of the food web (82), therefore exposed to lower mercury accumulation as compared to larger predatory fish, which are deemed to contain higher levels of methylmercury (83).

Amongst patients from two American prospective cohorts (51,529 males and 121,700 females), no clinically relevant associations were found between toenail mercury accumulation and higher risk of coronary heart disease, stroke, or total cardiovascular disease (80). Equally important, that study showed that higher fish consumption (≥ 2 servings/week) did not increase the likelihood of cardiovascular events based on toenail mercury concentrations, and that lower fish consumption (< 1 serving/week) did not reduce it (80). Such results contradict the assumption that mercury-induced cardiovascular disease is caused by general fish consumption.

The Prevención con Dieta Mediterránea (PREDIMED) trial, one of the largest prospective RCTs investigating the effects of a Mediterranean diet ever conducted, which recruited 7,477 adults at high risk for cardiovascular disease at baseline (84), showed that mercury exposure from regular fish consumption does not seem to increase cardiovascular disease risk (85). A nested case–control study examining a subpopulation (*n* = 147) of the PREDIMED trial who had eventually been diagnosed with cardiovascular disease along the course of the trial showed that toenail mercury concentrations detected in that subpopulation were comparable to age- and sex-matched controls (*n* = 267) (85).

## 5. The impact of sardine consumption on the omega-3 index

The omega-3 index (O3I), defined as the sum of EPA and DHA in the erythrocyte membrane, expressed as weight percentage of total fatty acids, has been proposed as a risk biomarker for coronary artery disease, particularly helpful following sudden cardiac arrest (86, 87). A meta-analysis (88), investigating 10 cohort studies found an average O3I of 6.1% ± 2.1% across the population included in the studies, with an O3I > 8% identified as protective against coronary heart disease mortality. Moreover, with median first and fifth quintiles at 4.2 vs. 8.3% respectively, the authors found that an O3I < 4% was a higher risk cut-off point (88). Other studies have proposed an O3I > 4 and < 8% as intermediate cut-off points for coronary heart disease risk, sudden cardiac death in particular (89, 90).

Results from the Canadian Health Measures Survey have shown that the average O3I in Canadians aged 20–79 years included in the survey was 4.5%, and that less than 3% of the sample population had a protective O3I of ≥ 8% (89). It is worth mentioning however that the data reported in that study was not obtained through erythrocyte membrane fatty acid chromatographic analyses, but instead indirectly *via* food diary analyses.

The Mediterranean Diet has traditionally been recognised as featuring moderate to high levels of n-3 PUFA, and the incidence of sudden cardiac death appears to be lower in Spain as compared to some other countries (91). Interestingly, a cross-sectional study involving 198 Spanish patients at high cardiovascular risk, with mean age 66 years and a relatively high 0.9 g/d EPA + DHA intake, showed that the mean O3I was 7.1% (86). Combined, the aforementioned studies suggest careful consideration on reaching an optimal O3I even in a population known for their habitual intake of oily fish and nuts.

A study conducted in Norway demonstrated that first-time myocardial infarction-patients without sudden cardiac arrest (*n* = 185) had a higher O3I compared to out-of-hospital cardiac arrest patients suffering their first myocardial infarction (*n* = 14; 6.48% vs. 4.59%, respectively; *p* = 0.002) (87). The same study showed that a 1% increase in the O3I was associated with a 58% reduction in the risk of ventricular fibrillation.

Two recent RCTs have investigated the effects of prolonged sardine consumption on T2D sufferers (25, 26). In one study, 200 g of sardine per week for 12 months resulted in a 1.3% increment in the O3I (6.6 ± 1.2 to 7.9 ± 1.3%) (25), whilst the other study found an increase of 2.7% (5.3 ± 0.3 to 8.0 ± 0.4%) when the participants consumed 100 g of sardines 5 days a week for 6 months (26). Both studies independently revealed that habitual sardine consumption can switch the O3I from intermediate to lower risk of mortality associated with coronary heart disease (25, 26).

Sardine intake appears to increase the O3I in a similar way to the intake of salmon that was grown on feeds containing mainly fish oil or rapeseed oil (92). Likewise, the O3I increase promoted by sardine consumption appears to be similar to that of low-dose fish oil supplementation (< 1 g/d) (93). However, high-dose fish oil supplementation (>4 g/d) is clearly more potent in elevating the O3I (94) (Table 2).

**Table 2:** The effects of sardine interventions compared to other sources of omega-3 (fish oil supplementation and salmon) on the omega-3 index.

Study	Design	Population	Sample size of the intervention group	Protocol/supplementation	Duration	Omega-3 index (%)
Díaz-Rizzolo et al., 2021 (25)	RCT	Pre-diabetes and ≥ 65 yo	75	200 g of sardine per week plus a T2D-prevention nutritional plan	12 mo	6.64 ± 1.22 → 7.90 ± 1.33 (pre- and post-intervention)^*^
Balfegó et al., 2016 (26)	RCT	Drug-naïve patients with T2D	17	100 g of sardines 5 days a week plus a standard diet for T2D	6 mo	5.3 ± 0.3 → 8.0 ± 0.4 (pre- and post-intervention)*
Grenon et al., 2015 (94)	RCT	Patients aged 50 and older with lower-extremity Peripheral Artery Disease	40	4.4 g/d of fish oil	1 mo	5.2 → 9.2 (pre- and post-intervention)*
Ramprasath et al. 2013 (93)	RCT	Healthy volunteers	24	600 mg of n-3 PUFA from krill oil	1 mo	4.97 ± 0.69 → 7.20 ± 1.35 (pre- and post-intervention)*
Ramprasath et al. 2013 (93)	RCT	Healthy volunteers	24	600 mg of n-3 PUFA from fish oil	1 mo	4.96 ± 0.59 → 6.51 ± 0.97 (pre- and post-intervention)*
Roos et al., 2020 (92)	RCT	Healthy volunteers	17	2 portions/week of salmon grown on feeds containing mainly fish oil, in which the average portion size was 157.1 g and the EPA + DHA content of fillets was of 2.1 g/100 g	18 w	↑2.3%^#^
Roos et al., 2020 (92)	RCT	Healthy volunteers	17	2 portions/week of salmon grown on feeds containing mainly rapeseed oil, in which the average portion size was 157.1 g and the EPA + DHA content of fillets was of 0.9 g/100 g	18 w	↑2.0%^#^

* *p* < 0.001 for intragroup comparison.

# *p* < 0.01 compared to control group.

DHA, docosahexaenoic acid; EPA, eicosapentaenoic acid; PUFA, polyunsaturated fatty acids; RCT, randomised control trial.

## 6. Nourishment: Food vs. supplementation

Currently in the United Kingdom, food supplements and their use are regulated by the Department of Health and Social Care Nutrition Legislation, which employs the expertise of various agencies including the Medicines and Healthcare Products Regulatory Agency (MHRA) to ensure medicinal agents regulated by the United Kingdom are not contained in a food product, and the Food Standards Agency (FSA) which is responsible for policy on food safety, food labelling, genetically modified foods, novel foods and food hygiene (95, 96). In the United Kingdom, the governmental definition of food supplements is ‘*any food the purpose of which is to supplement the normal diet and which is a concentrated source of a vitamin or mineral or other substance with a nutritional or physiological effect, alone or in combination and is sold in dose form*’ (97).

In Europe supplements are regulated under the European Commission’s Directive 2002/46/EC and their definitions of supplementation align closely to those of the United Kingdom. Both bodies of legislation, in the United Kingdom and Europe, are very clear that supplements are intended to correct nutritional deficiencies and help individuals ascertain the necessary nutrients and not to be utilised as medicinal products exerting metabolic action or for the treatment or prevention of diseases (95–97).

It has been noted (98) however that society in general currently sees supplements being utilised with the intent of reducing the risk of chronic diseases such as cardiovascular disease, which is often brought on by an overconsumption of foods associated with sedentary lifestyles, rather than to treat vitamin and nutrient deficiencies as supplements had once done, with classical examples including scurvy and rickets (98–100). The consumption of food supplements to treat chronic diseases when no nutritional deficiency is present remains a matter of relevant debate.

The main reasons why most individuals consume supplements focus on maintenance or improvement of their health. Use of supplements empowers the individual, giving the sense they are actively taking measures to improve their health and longevity (98–100). It is estimated that nearly half of the population in most developed countries are taking supplements (101–103). In the United Kingdom, the 2016 Health Food Manufacturers’ Association (HFMA) National Survey reported that 59% of the adult population take food supplements (103). That was a 4% rise over the previous 2 years (102), and the Coronavirus pandemic marked an even greater increase with now over 70% of United Kingdom adults utilising supplements (103, 104).

Of those adults in the United Kingdom ingesting supplements, almost half of them (45%) consume them five times or more per week (103). Women in most countries are slightly more inclined to consume supplements than men and usage increases with age in both sexes (101–103). The consumption of n-3 PUFA and cod liver oil supplements in the UK is not as wide as multivitamin/mineral (MVM) supplements; however, 35% of the population regularly utilise them and their popularity increase with the age of the consumers (100).

In European countries, the EU Consumer Survey on Food Supplements 2022 reported that of those surveyed who had consumed supplements in the previous year, only 19% consumed n-3 PUFA or fish oils, whilst many consumed vitamin D (46%), vitamin C (36%), magnesium (33%) and MVMs (29%) (102). Of those who did consume n-3 PUFA or fish oil, 63% maintained a regular daily intake of the supplement as opposed to taking it occasionally or seasonally.

## 7. Sardines vs. n–3 PUFA supplements

Although sardines and oily fish in general are important sources of n-3 PUFA, patient compliance remains a challenge if n-3 PUFA intake is not followed. Interestingly, fish oil supplementation will not necessarily be the solution for all cases. A few studies have demonstrated that n-3 PUFA supplementation achieved by fish oil supplementation will not always significantly reduce the risk of major cardiovascular events, nor will it always be useful for the primary and secondary prevention of cardiovascular events, including all-cause mortality, cardiovascular mortality and cardiovascular events, coronary heart disease mortality, stroke, and arrhythmia (105, 106). A multicentre RCT recruiting 8,179 patients with confirmed cardiovascular disease or diabetes and under statin therapy found that daily supplementation with highly purified eicosapentaenoic acid ethyl ester (icosapent ethyl, Vascepa^®^; 4 g/d) was more effective in reducing the risk of cardiovascular events as compared to the placebo-receiving group (107). This strategy is considered a pharmacological intervention and should be preferably indicated for patients at high risk of cardiovascular disease, rather than targeted at the general population.

Whilst a more purified form of 3-PUFA along with removal of toxic contaminants is expected in fish-oil-based supplements (108), some level of oxidation is expected for n-3 PUFA in fish intake given its exposure to high temperatures, cooking and preparation methods. Both fried and baked oily fish increase bioactive oxidized n-3 PUFA products, mainly F-4 t-neuroprostanes derived from DHA (109). Cooked samples of oily fish also generate cholesterol oxidation products, in particular 7-ketocholesterol and cholestanetriol—the latter is the most cytotoxic cholesterol oxidation products (110). Moreover, cooked samples of oily fish contain more n-6 PUFA than raw samples, increasing n-6/n-3 PUFA ratios (110).

Besides cooking, the gastrointestinal system *per se* is another concern due to a profound effect on n-3 PUFA oxidation in both sardines and fish-oil-based supplements, limiting the n-3 PUFA bioavailability (111). Apparently however, such observed oxidation does not nullify the benefits of ingesting n-3 PUFA from fish as several studies have identified beneficial effects (112, 113). Thus, the beneficial effects of n-3 PUFA from fish consumption cannot be neglected for the overall population. Nevertheless, therapeutic strategies based on specific n-3 PUFA supplementation plans, or EPA alone in some cases, can be more useful in patients at higher risk of cardiovascular events and metabolic disease (114–118).

## 8. The bottom line: What is the recommendation?

According to the 2015–2020 Dietary Guidelines for Americans, 1 to 2 seafood meals per week may be included to reduce the risk of congestive heart failure, coronary heart disease, ischaemic stroke, and sudden cardiac death, especially when seafood replaces the intake of less healthy foods (23). Based on those guidelines, it is reasonable to recommend at least 1–2 servings of sardines per week aiming at cardiovascular benefits. In addition, as discussed earlier, a study observed cardiovascular benefits when participants consumed 100 g of sardines 5 days a week (26), but this amount and frequency tends to be unfeasible in the long term.

A personalised recommendation consisting of sardine consumption higher than the general guidelines of 1–2 portions weekly can be safely followed, depending on the individual’s acceptance and adherence to the diet plan. Noteworthy, a Science Advisory from the American Heart Association recommends n-3 PUFA (EPA + DHA or EPA-only) at a dose of 4 g/d as an effective and safe strategy for hypertriglyceridemia as monotherapy, or as an adjunct to other lipid-lowering drugs (119). An intake of sardines equivalent to 400 g daily would be necessary to reach this n-3 PUFA amount.

## 9. The issue of satiety

Whilst supplements are a convenient method of achieving a daily dose of n-3 PUFA, if not consumed with food, they are unlikely to achieve satiety the same way a portion of sardines or another oily fish would. Interestingly, several studies investigating the effects of n-3 PUFA and fish oil have demonstrated an increase in appetite in healthy and diseased individuals associated with their supplementation (99, 120, 121). Moreover, n-3 PUFA have been shown to possess orexigenic properties, increasing neuropeptide Y and decreased α-melanocyte-stimulating hormone and serotonin receptor activity in tumour bearing anorexic rats (122).

Satiation is regulated by the gut-brain axis in the form of visceral neural and blood-borne signals that reach the central nervous system to modulate orexigenic and anorexigenic responses. Mechanistically this occurs in the form of gut distension, content composition and rate of emptying, and also through chemoreceptors signalling nutrient composition by way of afferent signals *via* the vagus nerve to the nucleus of the solitary tract and subsequently to the lateral hypothalamic area. The consumption of supplements without food, or as a replacement for food, would fail to mechanistically modulate appetite in the same way food physically present in the alimentary canal does.

## 10. Conclusion

Only very few RCTs have investigated the effects of sardine consumption on human health. However, due to their nutritional composition, sardines may be considered a functional food and an adjuvant in the management of cardiometabolic diseases with a pro-inflammatory background. Sardines are an important source of n-3 PUFA in addition to components known for their cardioprotective effects, including calcium, potassium, magnesium, zinc, iron, taurine, and arginine. These nutrients in synergy are essential to modulate inflammation and oxidative stress related to the cardiovascular system and participate in cardiomyocyte and haemodynamic functions.

Notwithstanding the myriad of physiological interactions amongst micronutrients, amino acids and fatty acids discussed here, clinical wisdom must be exercised when making recommendations, as the nutrient amount across the food matrix does not have the same magnitude as therapeutic dosages (i.e., supplementation regimens).

## Author contributions

HS conceptualized the idea and drafted the first version of the manuscript. HS, TM, and AB contributed to the literature review, data gathering and discussion, and manuscript writeup. All authors have read and approved the final version of this manuscript.

## Funding

HS has been supported by Coordenação de Aperfeiçoamento de Pessoal de Nível Superior (CAPES), Brazil.

## Conflict of interest

The authors declare that the research was conducted in the absence of any commercial or financial relationships that could be construed as a potential conflict of interest.

## Publisher’s note

All claims expressed in this article are solely those of the authors and do not necessarily represent those of their affiliated organizations, or those of the publisher, the editors and the reviewers. Any product that may be evaluated in this article, or claim that may be made by its manufacturer, is not guaranteed or endorsed by the publisher.
